# Association of trace elements abnormalities with thyroid dysfunction

**DOI:** 10.4314/ahs.v21i3.56

**Published:** 2021-09

**Authors:** Maha M Al-Bazi, Taha A Kumosani, Abdulrahman L Al-Malki, Kurunthachalam Kannan, Said S Moselhy

**Affiliations:** 1 Department of Biochemistry, Faculty of Science, King Abdulaziz University (KAU), PO Box 80203, Jeddah, Saudi Arabia; 2 Experimental Biochemistry Unit, King Fahd Medical Research Center, King Abdulaziz University (KAU), Jeddah, Saudi Arabia; 3 Production of Bio-products for Industrial Applications Research Group, King Abdulaziz University (KAU) Jeddah, Saudi Arabia; 4 Bioactive natural products Research Group, KAU, Jeddah, Saudi Arabia; 5 Department of Environmental medicine, University School of Medicine, New York, NY10016, USA; 6 Department of Biochemistry, Faculty of Science, Ain Shams University, Cairo, Egypt

**Keywords:** Trace metals, heavy elements, thyroid dysfunction

## Abstract

**Background:**

The metabolic pathways can be affected by dysregulation in thyroid hormone levels which in turn can arise from environmental chemical exposure. This study investigated the association of selected trace elements with thyroid disorders in a Saudi population.

**Methods:**

Urine samples collected from 100 participants (50 thyroid disorder patients and 50 controls) were analyzed to determine trace elements using inductively coupled plasma-mass spectrometer. Non-parametric Mann-Whitney Test, were used to examine the association between socio-demographic as well as clinical characteristics of thyroid profile levels (T3, T4 and TSH) and urinary trace element concentrations.

**Results:**

Urine from patients with thyroid disorders had significantly higher concentrations of Ni, Cu, and Cd (p-values <0.0005). In contrast, urinary Cr and Zn (p-values <0.013 and 0.005) were low in thyroid patients compared to the control.

**Conclusion:**

First study to report urinary trace element levels showed a possible link between thyroid disorders and trace element exposure which reflect the environmental pollution..

## Introduction

In modern societies, people are exposed to many environmental chemicals. These chemicals accumulate in human body and adversely affect health^1^. Biomonitoring has been used in the assessment of human exposure to environmental chemicals^2^. Chemicals found in air, water, soil, dust, food or even consumer products are referred to as environmental chemicals^3^. Over 300 environmental chemicals or their metabolites could be assessed in human samples such as urine, blood, serum or breast milk^4^. The group of environmental chemicals that have an effect on endogenous hormones' synthesis, secretion, transportation, metabolism, binding or elimination are called endocrine-disrupting chemicals (EDCs)^5^. Environmental chemicals can alter thyroid hormones. Many diseases can be caused by thyroid gland dysfunction^6^. Hypothyroidism, hyperthyroidism, Grave's Disease, Hashimoto's Disease and iodine deficiencies are the most prevalent thyroid disorders^7^.

Thyroid hormone profiles, including TSH, T3 and T4, are important indicators of thyroid functions. Hypothyroidism or hyperthyroidism, are leading common endocrine disorders in the U.S.^8^ The common risk factors of hyper or hypothyroidism include age, sex, radiation, chemotherapy and autoimmune disease^9^.

A few studies reported exposure to heavy metals and thyroid disorders. Blood mercury was reported to be inversely associated with T3 and T4 and urinary cadmium was positively associated with T3 and T4^10^.

The study using data from the NHANES 2007–2008, reported that there was no association between thyroid hormones and blood lead levels. However, using the same NHANES population, suggested that lead in both blood and urine was associated with decreased total thyroxine (TT4). Among females, they reported a positive association between lead and free thyroxine (FT4) and no association between cadmium and any of the thyroid hormones. The existing literature has provided suggestive evidence of a relationship between exposures to heavy metals and thyroid diseases^11^,^12^. Using self-reported thyroid diseases status (thyroid dysfunction), it was found a positive relationship between thyroid problems and urinary cadmium, cobalt, lead, and tungsten, although the association were not statistically significant. The self-reported disease status may not accurately measure subclinical hyper/hypothyroidism. In this study, we used serum thyroid hormone levels measured to clinically define thyroid diseases and measured urinary concentrations in the same trace and heavy metals (Cr, Mn, Co, Ni, Cu, Zn, As, Cd, Ba, Ti, Pd and U) to examine the relationship between thyroid homes and heavy metal accumulation.

## Study population and methods

This is a community-based clinical control study involving the analysis of urinary trace elements (n=100, 50 thyroid disorder cases and 50 controls). Urine samples were collected from participants of ages ranging from 20 to 79 years, who were residents of Jeddah, Saudi Arabia, during the period January 2017-January 2019. A survey questionnaire was used to collect information regarding thyroid status, age, gender, nationality, smoking status, and education level. Urine from all cases were collected from King Abdulaziz Hospital in Jeddah. Thyroid disorder cases were recruited during their routine checkup visit at endocrinology clinic and were identified through a positive diagnosis by a physician from the analysis of T3, T4 and TSH and then confirmed based on the medications taken to manage the condition. The controls were randomly selected from a non-thyroid represented the target population in terms of sociodemographic distribution. The control group was recruited through vluntary participation, at an approximate 1:1 ratio and confirmed free of thyroid diseases, T3, T4 and TSH. The controls were recruited from residents of Jeddah, Saudi Arabia. Spot urine samples were collected in polypropylene (PP) conical tubes from cases and controls, after the administration of the questionnaire. Institutional Review Board approvals of both King Abdulaziz University and the Ministry of Health, Directorate of Health Affairs, Medical studies and Research Department in Jeddah, were obtained before sample collection. All urine samples were stored in a freezer at -20 °C until chemical analysis.

## Determination of serum Free T3 and T4.

The Free T3 or T4 method is a homogeneous, sequential, chemiluminescent immunoassay based on luminescent oxygen channeling immunoassay (LOCI) technology. The LOCI reagents include two synthetic bead reagents and a biotinylated anti-T3 sheep monoclonal antibody. The first bead reagent (Chemibeads) is coated with diiodothyronine (T2), a naturally occurring, weaker binding analog of T3 or T4 and contains chemiluminescent dye. The second bead reagent (Sensibeads) is coated with streptavidin and contains a photosensitizer dye. In a first step, the sample is incubated with a biotinylated antibody which allows T3 ot T4 from the sample to saturate a fraction of the biotinylated antibody that is directly related to the free T3 concentration. In a second step, T2 chemibeads are added and from bead/biotinylated antibody immunocomplexes with the non-saturated fraction of the biotinylated antibody. Sensibeads are then added and bind to the biotin to form bead pair immunocomplexes. Illumination of the complex at 680 nm generates singlet oxygen from Sensibeads which diffuses into the Chemibeads, triggering a chemiluminescent reaction. The resulting signals are measured at 612 nanometer (nm) and are an inverse function of the FT3 concentration in the sample.

Determination of Thyroid Stimulating Hormone (TSH) TSH method is a homogeneous, sandwich chemiluminescent immunoassay based on LOCI technology. The sample is incubated with biotinylated antibody and chemibeads to form bead-TSH-biotinylated antibody sandwich. Sensibeads are added and bind to the biotin to form bead pair immune complexes. Illumination of the complex at 680 nm generates singlet oxygen from Sensibeads which diffuses into the Chemi beads, triggering a chemiluminescent reaction. The resulting signals are measured at 612 nm and are a direct function of the TSH concentration in the sample.

## Analysis of trace elements

Urine samples were digested by Top wave Analytic Jena microwave digestion system using ultra-pure nitric acid. About 1.5 ml urine was put into the DAP 60 digestion vessels of 60 ml capacity. Seven ml of nitric acid was added and mixed carefully. A blank without the sample was also analyzed through the complete procedure. After at least 30 minutes the vessel was closed and heated in the microwave oven as shown in Table 1.

**Table 1:** Microwave oven program used in the analysis of urinary trace elements

Step	Temperature (°C)	Pressurebar	Rampmin	Timemin	Power%
1	180	50	5	5	90
2	190	50	1	15	90

The interior walls of the vessels were washed with ultra-pure de-ionized (18 MΩcm-1) water and the vessels were swirled throughout the digestion to keep the wall clean and prevent the loss of the samples. Thereafter, the solution was transfer into a plastic tube and diluted to 25 ml in ultra-pure de-ionized water. The samples were analyzed with inductively coupled plasma-mass spectrometer, Thermo Fisher scientific) in central laboratory, college of science, King Saud University, Riyadh, KSA. The external calibration was carried out busing multi-elements standard. Table 2 provides information on operating conditions of ICP-MS.

**Table 2:** ICP-MS operating conditions used in the analysis of trace elements in urine

Parameters	Value/ Condition
RF frequency	40 MHz
RF Power	1548.6 W
Pirani Pressure	1E+2 mbar
Penning Pressure	9.549E-8 mbar
Detector Counting Voltage	1750 V
Detector Analog Voltage	-1825 V
Plasma gas flowAr, 99.997	13.84 L/min
Auxiliary gas flowAr, 99.997	0.8 L/min
Nebulizer gas flowAr, 99.997	0.9 L/min
Sampler and Skimmer cone	Nickel
Mode of operation	Standard mode (STD)
Sample Uptake	30 s
Peristaltic Pump Rate	40 rpm
Nebulizer	Glass concentric type
Spray Chamber	Quartz, Cychronic type
Spray Chamber Temperature	-20 °C
Injector	Quartz, 2.5 mm ID
Torch	Two concentric quartz tubes
Sample tubing	Standard 0.508 mm i.d.
Drain tubing	Standard 1.29 mm i.d.
Dwell Time	0.01 s
Number of Replicates	3
Rinse Time	30 s
Resolution m / z	238 amu
Isotope ratio precision CeO / Ce	<3 %
Short-term stability	< 3% RSD

## Statistical Analysis

All analyses, including the Pearson's correlation, were conducted by SPSS 20 (IBM SPSS Statistics, ver. 20.0 Armonk, NY, USA). non-parametric Mann-Whitney Test was used. (It was reported in abstract; mention also the evaluation of p-value. It was reported in the results section the Kruskal-Wallis test was used

## Results

### Demographic analysis

The Kruskal-Wallis test was used to compare independent groups (control and thyroid disorders group). A Mann-Whitney U test was used to test for pairwise comparisons (Table 3). The mean age of the control group participants was 23.1 ± 6.53 years, and the mean age of the participants in thyroid group was 45.2 ± 11.3 years. There was evidence of a statistically significant difference (p= 0.0001) in the continuous outcome variable (age and BMI) between thyroid group and control group. Moreover, there was a statistically significant difference (p=0.0001) in the continuous outcome variable age between those diabetic patient group and thyroid patients' group. Table (4) showed that, there was a significant increase in TSH level (p =0.014) in thyroid disorders group (7.54±19.5 µIU/ml) as compared to the control group (1.8±0.899 µIU/ml), respectively. There was a significant increase in T4 level (p =0.000) in thyroid disorders group (12.01±3.85 pmol/l as compared to the control group (14.3±2.33 pmol/l.

**Table 3:** Demographic analysis of studied groups

Variables		Thyroid patients		Chi-Square Tests	Controls	
		n=50	100%	p-value	n=50	100% (total)
**Gender**	**Male**	5	23.8	.318	1	4.8
	**Female**	45	34.9		49	38
**Age**	**20–30 years**	7	13.2	*<0.0005*	44	83
	**31–40 years**	10	50		4	20
	**41–50 years**	19	63.3		2	6.7
	**51–60 years**	11	42.3		0	0
	**>60 years**	3	14.3		0	0
**BMI**	**Underweight**	0	0	*0.106*	9	100
	**Normal**	12	30		23	57.5
	**Overweight**	17	34		12	24
	**Obese**	21	41.2		6	11.8
**Marital** **Status**	**Single**	5	10.4	*<0.0005*	39	81.2
	**Married**	41	47.7		11	12.8
	**Widow**	1	10		0	0
	**Divorced**	3	50		0	0
**Education**	**No schooling**	4	25	*<0.014*	0	0
	**General** **education**	30	46.2		2	3.1
	**Higher** **Education**	16	23.2		48	69.6
**Smoker**	**No**	50	35	*0.054*	48	33.6
	**Yes**	0	0		2	28.6
**Thyroid**	**No**	0	0		50	51
	**Hypothyroidism**	42	95.5		0	0
	**Hyperthyroidism**	8	100		0	0
**Heart** **disease**	**No**	49	34.8		50	35.5
	**Yes**	1	11.1		0	0
**Blood** **pressure**	**No**	43	39.8		50	46.3
	**Hypertension**	7	16.7		0	0

BMI: body mass index.** Significant at level 0.01.

**Table 4:** Thyroid hormone levels among the study populations

Thyroid function Parameters	Thyroid disorders group n = 50	Control group n =50
Thyroid stimulating hormone (TSH) µIU/ml	7.54±0.95a	1.8±0.89
T3 (pmol/l)	3.7± 0.27	4.82±1.55
T4 (pmol/l)	10.01±1.85	14.3±2.33

T3: triiodothyronine; T4: thyroxine.

n = number of participants in each group

**a** significant versus control group.

Data in table (5) investigated the correlation of thyroid function parameters and trace elements concentrations within thyroid disorders group. In the current study, there was significant positive correlations between TSH level and Ba (r= 0.341, P=0.015). While there were non-significant correlations between T3 and T4 level and all trace elements. In the present study, there was a significant positive correlations between Co and Mn (r= .734, p= 0.0005), Ni (r= 0.864, p= 0.0005), Zn (r= 0.484, p= 0.0005), As (r= 0.526, p= 0.0005), Cd (r= 0.451, p= 0.001), Pd (r= 0.587, p= 0.0005), U (r= 0.801, p= 0.0005), and Tl (r= 0.339, p= 0.016). While there was a significant negative correlation with Cu (r= -0.306, p= 0.31). Interestingly, there was a significant positive correlation between Cr and Cu (r= 0.521, p= 0.000). Regarding Ni, there was a significant positive correlations versus Mn (r= 0.809, p= 0.0005), Cd (r= 0.519, p = 0.000), As (r= 0.514, p= 0.000), Tl (r= 0.287, p= 0.043), Pd (r= 0.622, p= 0.000), and U (r = 0.554, p= 0.000). While there was a significant negative correlation with Cu (r = -0.535, p= 0.0005). There were significant negative correlations between Cu versus Zn (r= -0.480, p= 0.000), Cd (r= -0.584, p= 0.000), Pd (r= -0.480, p= 0.000), and Mn (r= -0.490, p= 0.000). Arsenic exhibited a significant positive correlations versus Cd (r= 0.343, p= 0.015), Ba(r= 0.404, p= 0.004), Ti (r= 0.619, p= 0.000), Pd (r= 0.384, p= 0.006), U (r= 0.392, p= 0.005) and Mn (r= 0.511, p= 0.000). Regarding Cd, there was a significant positive correlations versus Ba (r= 0.418, p= 0.003), Pd (r= 0.581, p= 0.000), U (r= 0.395, p= 0.000), and Mn (r= 0.306, p= 0.031). Barium showed a significant positive correlation versus Ti (r= 0.466, p= 0.001) and U (r= 0.417, p= 0.03). Finally, uranium showed a significant positive correlation versus Ti (r= 0.315, p= 0.026), Pd (r= 0.543, p= 0.000) and Mn (r= 0.358, p= 0.006).

**Table 5:** Urinary trace elements concentrations (ng/mL; ppb) among the study population Mean ±SD.

n=50		Mean ±SD
**Cr**	**Thyroid patients**	6.00±1.30 a
	**Controls**	35±2.63
**Mn**	**Thyroid patients**	3.87±0.21^a^
	**Controls**	2.88±0.1
**Co**	**Thyroid patients**	0.314± 0.01
	**Controls**	0.351±0.011
**Ni**	**Thyroid patients**	15.02±1.1 a
	**Controls**	9.61± 0.81
**Cu**	**Thyroid patients**	101.4 ±4.5 a
	**Controls**	44.8±2.1
**Zn**	**Thyroid patients**	8.26 ±0.98 a
	**Controls**	15.48 ±1.4
**As**	**Thyroid patients**	0.972±0.04 a
	**Controls**	1.17 ±0.06
**Cd**	**Thyroid patients**	0.024± 0.001 a
	**Controls**	0.004± 0.003
**Ba**	**Thyroid patients**	0.694± 0.001 a
	**Controls**	0.256± 0.004
**Tl**	**Thyroid patients**	0.016± 0.001
	**Controls**	0.014 ±0.005
**Pd**	**Thyroid patients**	2.05± 0.01 a
	**Controls **	1.37± 0.003
**U**	**Thyroid patients**	0.16 ±0001 a
	**Controls**	0.22± 0.01

n = number of participants in each group; ppb: part per billion.

a significant versus control group.

Using Univariate logistic regression, table (6) revealed that the trace elements Cr, Cu, Zn, Pd, and Ba have positive significant effects on the thyroid function, p-value = 0.004, 0.005,0.019, 0.031 and 0.002 and OR= 1.06,1.06, 1.059, 3177.26 and 7428.9; respectively. Moreover, the trace elements Co, As, Ti, U, and Mn, OR= 0.159, 0.673, not detected (nd), 0.228, and 0.633; respectively, have negative significant effects on the thyroid function, p-value=0.0005, 0.0005, 0.09, 0.0005 and 0.001; respectively. That is, being a thyroid disorder patient is dependent on the increase of (Cr, Cu, Zn, Pd, and Ba) and the decrease of Co, As, Tl, U, and Mn.

**Table 6:** Comparison of the concentrations of trace elements (ppb) in thyroid disorder participants and controls group

Test	Cr	Mn	Co	Ni	Cu	Zn	As	Cd	Ba	Tl	Pd	U
**Mann-** **Whitney U**	894.500	785.000	1154.500	523.000	677.000	845.500	1204.0	754.0	1029.5	1243.0	988.0	1223.5
**Sig. (2-tailed)**	.013*	.001*	.510	.000***	.000***	.005*	.751	.000***	.120	.961	.068	.851

ppb: part per billion

* significant at p < 0.05 in contrast to the control.

** significant at p < 0.01

*** significant at p < 0.001 in contrast to the control

## Discussion

Thyroid activity can influence chromium levels, the low thyroid function can increase insulin secretion leading to chromium loss^13^. In this study, major reductions in urinary concentrations of Cr were observed in patients with hypothyrodism. Our results are consistent with the results previously reported^14^,^15^. Gastrointestinal absorption of Cr may be reduced in thyroid patients^16^. Manganese is an essential constituent of some enzymes such as pyruvates carboxylase and arginase and a stimulus of other enzymes, including phosphoenolpyruvate carboxykinase and glutamine synthetase. The activated enzymes of manganese play important roles in carbohydrates, amino acids and cholesterol metabolism. Hypothyroid patients appear to havactual copper increase, whereas hyperthyroid patients have a deficiency of copper^17^. In the present study, there was a significant increase of the urinary Cu in hypothyroidism patients as compared to the control healthy subjects and significant positive association between the Cu concentrations and thyroid disease by logistic regression analysis. Likewise, in contrast to controls Sinha and his colleagues have found a significant increase in Cu concentration in hypothyroidism patients. Most plasma Cu is bound to ceruloplasmin (CP), the major copper-carrying protein in the blood, and only a small part is tied to plasma albumin. The increase in serum and decrease in urinary Cu can therefore be a result of hyperthyroidism due to elevated CP. Plasma CP levels in patients with hyperthyroidism were also confirmed to be increased. It was suggested that increased ceruloplasmin and slow excretion of Cu from the body may explain high serum Cu levels in hyperthyroidism^18^. Thyroid hormones control the metabolism of zinc by influencing the absorption and excretion of zinc. On the other hand, zinc deficiency affects the function of the thyroid. In fact, thyroidzinc relationship is complex and includes synthesis and mode of actions^19^. However, the effects of zinc on the level of thyroid hormones and the thyroid gland in general are still unknown. The results of present study are consistent with the results of previous studies^20^. It was reported that Serum Zn is primarily combined to and transported by albumin. Serum albumin level in hyperthyroid state decreases, decreases Zn-albumin complex may lead to increasing Zn serum ultrafiltration, accelerating Zn excretion into urine^21^. In the present study, logistic regression analysis revealed a significant relationship between zinc levels and thyroid function. Such results are consistent with Rezaei and his collaborators who have reported that in hyperthyroidism and hypothyroidism, the effects of Cr, Co, Pb, Cu, Zn and Cd were important, while in thyroid cancer patients, the effects of Cr, Cd and Pb were small^22^.

In the present study, there was significant increase in thyroid disorder group as compared to the control healthy group. The current study result is in agreement with the results of some previous studies. A similar population detected no significant relationship between the variables^23^. A negative Pd-total thyroxin relationship, as well as the lack of significant Pb-TSH, T3 and T4 association. It was found a similar absence of a significant relationship between Pb and thyroid hormone concentration^24^. However, it was noted the missing connection between concentrations of thyroid hormones and levels of Pb in people. In women, however, a positive relation between Pb and T3 and a negative one between Pb and TSH was reported^25^. In the current study, tere was a significant increase in Cd level in thyroid disorder group as compared to the control group. Correspondingly, the literature available also includes some evidence that indicate a significant increase in Cd level in the thyroid disorders patient relative to the stable control group and a negative but not significant cadmium effect on the thyroid gland^26^. It was reported that, the animal cadmium exposure and showed decreased T4 and T3 concentrations. The proposed mechanism explaining the mentioned effect could include e.g. inhibiting thyroxin synthesis, or release, an altered T4 deiodination process due to 5-deiodinase inhibited activity. It was demonstrated significantly higher TSH levels in Cd-treated rats than in the control group. Christensen et al. have identified a negative correlation between blood cadmium and TSH and the positive correlation between urinary cadmium concentration and T4 and T3 concentrations^27^. Urinary thallium and barium were associated with decreased T4 (both) and T3 (barium)^28^. It was identified a significant association between arsenic concentration and serum TSH concentration. Arsenic was associated with a dose-dependent increase in TSH. For arsenic these findings were consistent with recent experimental studies where arsenic inhibited enzymes involved in thyroid hormone synthesis and signaling^29^. This could explain the results of the current study, which revealed no significant association between As levels and the thyroid functions.

We concluded that, significantly high urinary levels of Cu, Zn and Cr, and a significant decrease in Mn in comparison with healthy people. In addition, there was a significant increase in Mn, Co and Ni in thyroid patients, while there was a significant decrease in Cr and Zn compared to healthy subjects. Therefore, the imbalance of the studied metal levels may play an important role in the pathogenesis of diabetes and thyroid disorders.

## Data Availability

The authors confirm that the data supporting the findings of this study are available within the article.

## References

[1] Badran M, Morsy R, Soliman H, Elnimr T (2016). Assessment of trace elements levels in patients with type 2 diabetes using multivariate statistical analysis. Journal of Trace Elements in Medicine and Biology.

[2] Bahn R S, Burch H B, Cooper D S, Garber J R, Greenlee M C, Klein I, Laurberg P, Mcdougall I R, Montori V M, Rivkees S A (2011). Hyperthyroidism and other causes of thyrotoxicosis: management guidelines of the American Thyroid Association and American Association of Clinical Endocrinologists. Thyroid.

[3] Balenko N, Tsymbaliuk S, Chernichenko I, Ostash O (2017). The role of carcinogenic metals in the formation of thyroid cancer morbidity in the population. Environment and Health.

[4] Baltaci A, Mogulkoc R, Belviranli M (2013). L-thyroxine-induced hyperthyroidism affects elements and zinc in rats. Bratislavske Lekarske Listy.

[5] Chen A, Kim S S, Chung E, Dietrich K N (2013). Thyroid hormones in relation to lead, mercury, and cadmium exposure in the National Health and Nutrition Examination Survey, 2007–2008. Environmental Health Perspectives.

[6] Chen C, Wang N, Nie X, Han B, Li Q, Chen Y, Zhai H, Zhu C, Chen Y, Xia F (2016). Blood Cadmium Level Associates with Lower Testosterone and Sex Hormone-Binding Globulin in Chinese men: from SPECT-China Study, 2014. Biological Trace Element Research.

[7] Chen SY, Hwang JS, Sung FC, Lin CY, Hsieh CJ, Chen PC, Su TC (2017). Mono-2-ethylhexyl phthalate associated with insulin resistance and lower testosterone levels in a young population. Environmental Pollution.

[8] Ertek S, Cicero A F, Caglar O, Erdogan G (2010). Relationship between serum zinc levels, thyroid hormones and thyroid volume following successful iodine supplementation. Hormones.

[9] Esteban M, Castaño A (2009). Non-invasive matrices in human biomonitoring: a review. Environment International.

[10] Farid S M (2012). The association between serum glucose and serum lead and selected trace elements in type 2 diabetes mellitus patients in Jeddah, Saudi Arabia. Medical Journal of Islamic World Academy of Sciences.

[11] Farid S M, Abulfaraj T G (2013). Trace mineral status related to levels of glycated hemoglobin of type 2 diabetic subjects in Jeddah, Saudi Arabia. Medical Journal of Islamic World Academy of Sciences.

[12] Feng W, Cui X, Liu B, Liu C, Xiao Y, LU W, Guo H, HE M, Zhang X, Yuan J (2015). Association of urinary metal profiles with altered glucose levels and diabetes risk: a population-based study in China. PloS One.

[13] Kasimbaltaci A, Belviranli M (2013). Serum levels of calcium, selenium, magnesium, phosphorus, chromium, copper and iron-their relation to zinc in rats with induced hypothyroidism. Acta Clinica Croatica.

[14] kazi T G, Kandhro G A, Afridi H I, Kazi N, Baig J A (2010). Interaction of copper with iron, iodine, and thyroid hormone status in goitrous patients. Biological Trace Element Research.

[15] Khadem-ansari M H, Ghodratizadeh S, Hajizadeh R, Jangi H, Manafi M, Rasmi Y (2017). Serum zinc and copper variations in patients with hyperthyroidism. New Armenian Medical Journal.

[16] Khalid S, Samia A, Muneera A, Patan M, Abdulla M (2019). The Prevalence of Hypothyroidism in Patients with Type 2 Diabetes Mellitus in Saudi Community based Hospital a Retrospective Single Centre Study. Archives of Diabetes & Obesity.

[17] Shukla GS, Chandra S V (1982). Chandra Effects of manganese on carbohydrate metabolism and mitochondrial enzymes in rats. Acta Pharmacol Toxicol (Copenh).

[18] Mendy A, Gasana J, Vieira E R (2012). Urinary heavy metals and associated medical conditions in the US adult population. International Journal of Environmental Health Research.

[19] Mendy A, Gasana J, Vieira E R (2013). Low blood lead concentrations and thyroid function of American adults. International Journal of Environmental Health Research.

[20] Nie X, Chen Y, Chen Y, Chen C, Han B, Li Q, Zhu C (2017). Lead and cadmium exposure, higher thyroid antibodies and thyroid dysfunction in Chinese women. Environmental Pollution.

[21] Padilla MA, Elobeid M, Ruden D M, Allison DB (2010). An examination of the association of selected toxic metals with total and central obesity indices: NHANES 99-02. International Journal of Environmental Research and Public Health.

[22] Pan WC, Seow W J, Kile M L, Hoffman E B, Quamruzzaman Q (2013). Association of low to moderate levels of arsenic exposure with risk of type 2 diabetes in Bangladesh. American Journal of Epidemiology.

[23] Pirahanchi Y, Jialal I (2018). Physiology, Thyroid, Stimulating Hormone (TSH).

[24] Porta M, Puigdomènech E, Ballester F, Selva J, Ribas-fitó N, Llop S, López T (2008). Monitoring concentrations of persistent organic pollutants in the general population: The international experience. Environment International.

[25] Prete A, Paragliola R M, Corsello S M (2015). Iodine supplementation: usage “with a grain of salt”. International Journal of Endocrinology.

[26] Swanson K, Harmon D, Jacques K, Larson B, Richards C, Bohnert D, Paton S (2000). Efficacy of chromium-yeast supplementation for growing beef steers. Animal Feed Science and Technology.

[27] Talebi S, Ghaedi E, Sadeghi E, Mohammadi H, Hadi A, Clark C C, Askari G (2019). Trace Element Status and Hypothyroidism: A Systematic Review and Meta-analysis. Biological Trace Element Research.

[28] Christensen KL (2013). Metals in blood and urine, and thyroid function among adults in the United States 2007-2008. Int J Hyg Environ Health.

[29] Wang YX, Pan A, Feng W, Liu C, Huang LL, Ai SH, Zeng Q, Lu WQ (2019a). Variability and exposure classification of urinary levels of non-essential metals aluminum, antimony, barium, thallium, tungsten and uranium in healthy adult men. Journal of Exposure Science & Environmental Epidemiology.

